# Galactography is not an obsolete investigation in the evaluation of pathological nipple discharge

**DOI:** 10.1371/journal.pone.0204326

**Published:** 2018-10-08

**Authors:** Aleksandr Istomin, Amro Masarwah, Marja Pitkänen, Sarianna Joukainen, Anna Sutela, Ritva Vanninen, Mazen Sudah

**Affiliations:** 1 Department of Clinical Radiology, Diagnostic Imaging Centre, Kuopio University Hospital, Kuopio, Finland; 2 Department of Plastic Surgery, Surgical Division, Kuopio University Hospital, Kuopio, Finland; 3 School of Medicine, Clinical Radiology, Institute of Clinical Medicine, University of Eastern Finland, Kuopio, Finland; University Hospital Zurich Department of Diagnostic and Interventional Radiology, Zürich, SWITZERLAND

## Abstract

**Purpose:**

To evaluate the malignancy rate and diagnostic performance of galactography in patients with pathological nipple discharge (PND) after negative clinical breast examination, mammography and ultrasound.

**Materials and methods:**

We retrospectively evaluated all galactograms obtained between January 2006 and December 2014 in women with PND. Galactographic findings were classified into 6 groups according to a modified Galactogram Image Classification system (GICS) to comply with the breast imaging reporting and data system classification. Observers were blinded to the final histology and clinical outcome at the time of analysis. MRI was performed as a problem solving ancillary examination. Imaging findings, pathological diagnosis and follow-up data were evaluated. The diagnostic performance of MRI and technically successful galactography in the detection of neoplastic or risk lesions were separately calculated.

**Results:**

A total of 146 patients with PND (mean age, 51.5 years; range, 17–93) were examined. Malignant lesions were detected in only 4 patients (2.7%) and risk-lesions in 5 patients (3.4%). Only one low-grade ductal carcinoma in situ was missed by galactography (GICS 1) and MRI. MRI examinations were performed in 21 (14.4%) patients; one of these patients (4.8%) had a malignant finding (GICS 0), two (9.5%) had risk-lesions (GICS 2 and 5). In the detection of neoplastic or risk lesions the sensitivity and specificity of galactography were 77.4% and 75.7% and of MRI 85.7% and 71.4%, consecutively.

**Conclusion:**

The malignancy rate is negligible if clinical, mammography, ultrasound and galactography examinations are negative. Galactography remains a practical, valuable and cost-effective examination procedure. If galactography is technically unsuccessful, MRI should be considered as an additional ancillary tool to evaluate the possible etiology of symptoms, but the routine use of MRI in all patients cannot be justified.

## Introduction

Nipple discharge (ND) is a relatively common symptom in clinical practice but it causes both anxiety and discomfort to many women. It can be categorized as either physiological or pathological. Pathological nipple discharge (PND) is any unilateral, bloody, clear or serous exudate that spontaneously appears from a single orifice. While most of the causes behind PNDs are benign, such as duct ectasia, papilloma, or non-proliferative entities, the malignancy rate has been reported to be quite significant, ranging from 6–20% of cases [1–4].

The diagnostic workup of PND remains controversial. The common consensus is that after a full medical history and physical examination, mammography is the first-choice investigation technique for the evaluation of patients presenting with a PND [5–7]. Ultrasonography (US) is considered as a useful additional examination for the evaluation of possible mammographically occult lesions [7]. The cytological examination of nipple discharge has a poor accuracy and has not been found to possess any significant complementary diagnostic value [8]. Surgical duct excision remains the gold standard to provide a definitive histological diagnosis and exclude the possibility of underlying malignancies [9]. In contrast, the role of galactography has remained unclear. While some investigators consider it to be a useful, even recommended, method for the evaluation of PND, others have concluded that galactography can no longer be considered as acceptable in the modern world of multimodal breast imaging [10].

Magnetic resonance imaging (MRI) is a highly sensitive technique in the detection of breast malignancies [11,12]. Several studies have confirmed the additive value of MRI for the improved sensitivity of cancer detection and treatment planning in the evaluation of PND [10,13–15]. Nevertheless, there is still insufficient evidence to recommend the routine use of MRI in the evaluation of PND [16]. Recent reports evaluating the utility of MRI in the setting of negative mammography and /or US have concluded that in the case of inconclusive findings, an MRI examination is indicated [15,17,18] and furthermore that a negative MRI should obviate surgical duct excision in most patients [5,18].

However, the diagnostic work-up of pathological nipple discharge remains challenging and investigational strategies are controversial. The purpose of this study was to evaluate the malignancy rate in pathological nipple discharge after a negative clinical breast examination (CBE), mammography, US and galactography with breast MRI only being used as a supplementary problem-solving investigation. To achieve this aim, we evaluated retrospectively 151 consecutive patients with PND in our University hospital over a nine year period.

## Materials and methods

### Background

Patients from two screening centers, two district hospitals, and all primary health care centers within our catchment area of approximately 250,000 people are referred to our tertiary University hospital for consultation and treatment. Prior to referral, all patients older than 30 years of age had undergone a minimum of a two view mammography and whole breast US performed by radiologists with experience in breast radiology. According to national guidelines, mammography is not obligatory in younger patients and is conducted only if any of the triple assessment findings is malignant or highly suspicious for malignancy. Galactography is seldom performed outside of our hospital, but when done elsewhere, all possible images are accessible through the regional picture archiving and communication system (PACS). All images are routinely re-evaluated by a specialized breast radiologist and accordingly, additional mammography views, second look US or other investigations are performed as needed.

### Patients

The inclusion criteria for this study were as follows: the presence of PND; negative CBE; mammography (except for very young patients) and whole breast US having been performed by a breast radiologist and all considered as negative for malignancy as well as for the cause of PND; galactography was performed as a second line investigation; pathological confirmation of findings or a minimum of 2 years’ radiological follow-up. All galactographic examinations performed between January 2006 and December 2014 were retrospectively retrieved from the regional PACS system and the clinical data of these patients were also retrieved from the local digital archives. Altogether 151 consecutive patients were retrieved who had been evaluated with galactography during this period and presented with PND. Five of these patients were excluded since they had undergone mammography or US examinations after the galactography, i.e. the final number of patients included in the analyses was 146. This study was approved by the ethics committee of the Kuopio University hospital, and the need for written informed consent was waived by the same committee due to its retrospective nature.

### Data collection

All available patient medical records were reviewed and the following parameters were recorded and included in a database: age at exam, personal and family history of breast or ovarian cancer, type of nipple discharge, mammographic and sonographic findings, patient’s management, histopathological findings, final diagnosis, clinical outcomes and imaging follow-up information. The patients were then divided into two groups according to age: age ≤ 50 years and older than 50 years. Nipple discharge “colors” were classified into two groups: serous and sero-sanguineous/ bloody. All available clinical and/or breast imaging follow-up data was reviewed.

### Mammography and ultrasound

As a primary evaluation, in addition to prior standard mammographic digital craniocaudal (CC) and mediolateral-oblique projections of both breasts, a lateral and/or spot-compression views of the subareolar region of the symptomatic breast were obtained at the discretion of the performing radiologist. In addition, bilateral whole breast and axillary-region US imaging had been routinely performed. If mammography and US remained negative, galactography was performed.

### Galactography

Galactography was performed by cannulating the secreting duct with a blunt dedicated 30-gauge cannula (Argon/Athens/Georgia/USA) connected to a 1 ml syringe. A contrast agent (Visipaque 250 mg I/ml) was injected according to the patient’s own tolerance or resistance or until the contrast agents were observed to leak from the cannulated orifice. Subsequently, the patient was positioned for a CC view usually while the needle is still in place taped to the skin, and if the contrast concentration was deemed unsatisfactory in mammography, then an additional contrast injection was administered and the CC view repeated before the acquisition of the lateral projection. Magnification views were acquired as needed.

Galactographic findings were classified into 6 groups according to the modified Galactogram Image Classification system (GICS) [19] to comply with the breast imaging reporting and data system (BI-RADS) classification (Table 1). Two experienced breast radiologists with >20 years of experience in multimodal breast imaging evaluated and classified the galactographic images in consensus. Observers were blinded to the final histology and clinical outcome at the time of analysis.

**Table 1:** Modified Galactogram Image Classification system (GICS) interpretation guideline and the breast MRI protocol.

Galactogram interpretation	
Category	Description
**GICS 0**	Failed.
**GICS 1**	Normal.
**GICS 2**	Benign finding. This category includes: duct ectasia (>3 mm) with mild smooth walled changes in caliber. No ductal filling defects.
**GICS 3**	Pseudolesion.
**GICS 4a**	A macrodefect in ductal filling with duct ectasia is observed, obstruction of the duct with concave termination in the main duct or in a bifurcation with a segmental duct. Single microdefect in segmental/subsegmental duct.
**GICS 4b**	Multiple macrodefects in segmental/ subsegmental ducts. Peripheral or central smooth walled cystic single filling defect lesion with no contrast extravasation. Abrupt termination of main duct.
**GICS 4c**	A combination of micro- and macrodefects. Peripheral or central smooth walled cystic single filling defect lesion with extravasation of contrast. Multiple peripheral duct-stenoses.
**GICS 5**	Multiple microdefects with a moth-eaten appearance and ductal wall irregularities.

### MRI

MRI was performed as a problem solving ancillary examination selectively i.e. only in those patients if mammography, ultrasonography and galactography were non-informative, if the extent of the disease remained unclear or in patients where galactography technically failed and PND persisted. During the years 2006–2009, bilateral breast MRI was performed using a 1.5T scanner. After that period, all MRI examinations were performed with a 3T MRI scanner; 3.0T MRI scanner (Philips Achieva TX, Philips N.V., Eindhoven, The Netherlands) 7-element phased-array bilateral dedicated breast coil. The MRI protocol is presented in Table 2.

**Table 2:** Breast MRI protocol.

Sequence	TR/TE (ms)	in-plane resolution mm	Slice thickness (mm)	Scanning time
T1-FFE	shortest/2.3	0.48 × 0.48	0.7	6 min 11 s
T2-TSE	5000/120	0.6 × 0.6	2	3 min 20 s
STIR	5000 /60	1 × 1	2	5 min 40 s
T1 dynamic^a^	shortest/ shortest	0.96 × 0.96	1	58.5 s
DWI^b^	shortest /95	1.15 × 1.15	4	4 min 8 s

FFE, fast field echo; TSE, turbo spin echo; STIR, Short tau inversion recovery.

^a^eTHRIVE spectrally adiabatic inversion recovery (SPAIR) fat suppression; pre-contrast and six phases after the gadoterate meglumine (0.1 ml/kg, 3 ml/s) injection followed by a saline chaser.

^b^DWI, diffusion weighted echo planar imaging with five respective b factors (0, 200, 400, 600 and 800 s/mm2).

### Final diagnosis

Patients were considered to have a benign pathology if they remained clinically and radiologically asymptomatic during follow-up (minimum 2 years). Both invasive carcinomas and DCIS (ductal carcinoma in situ) were classified as malignant. Single papillomas and more diffuse papillomatosis were combined under the papilloma diagnosis. The “other benign lesions” group included duct ectasia and fibrocystic lesions. Lobular neoplasias, radial scars and atypical ductal hyperplasias were categorized as “risk-lesions”.

### Statistical analysis

Statistical analysis was performed using the SPSS statistical software package (IBM SPSS Statistics for Windows, Version 22.0. Armonk, NY: IBM Corp.). Sensitivity, specificity, overall accuracy and positive and negative predictive values were calculated. Positive and negative predictive values were calculated by using the Bayes formula. The 95% confidence intervals were calculated by using the Wilson no-continuity correction formula [20]. Differences between patient groups according to age (Over/under 50 yrs.), symptoms and findings were evaluated using the Chi-square test and were considered significant if the p-value was <0.05.

## Results

The mean age of patients was 51.5 years (range 17–93). A positive family history of breast or ovarian cancer was reported in 26 patients, one of whom had a high (>30%) risk of breast cancer. Five patients had a previous personal history of breast cancers, one in the contralateral and four in the ipsilateral breasts (3 invasive carcinoma and 2 DCIS), which had been treated with wide local excision and radiotherapy 1–11 years before their galactographic examinations.

Mammography was performed and interpreted as normal in 138 patients, while mammography was deemed unnecessary in 8 young patients. Ultrasound was performed in all patients. Blue-dye guided surgical excision was performed in 91 patients. Patient, ND characteristics and the histological verification data are presented in Table 3.

**Table 3:** Patients, nipple discharge characteristics and the histological verification data of the 146 patients included in this study.

		Total n (%)	Other benign n (%)	Papillomas n (%)	Malignant lesions n (%)	Risk lesions n (%)
**Age**	**≤ 50 yrs**	67 (45.9)	46 (68.7)	20 (29.9)	0 (0.0)	1 (1.4)
	**> 50 yrs**	79 (54.1)	34 (43.0)	37 (46.8)	4 (5.1)	4 (5.1)
**Nipple discharge**	**Bloody or serosanguineous**	62 (42,5)	31 (50.0)	26 (41.9)	1 (1.6)	4 (6.5)
	**Serous**	84 (57,5)	49 (58.3)	31 (36.9)	3 (3.6)	1 (1.2)
**Histological verification**	**Operation/biopsy**	97 (66,4)	31 (32.0)	57 (58.8)	4 (4.0)	5 (5.2)
	**Follow-up**	49 (33,6)	49 (100.0)	0 (0.0)	0 (0.0)	0 (0.0)

The malignant lesions detected at final histology were invasive carcinoma no special type (n = 2), invasive papillary carcinoma (n = 1) and low-grade DCIS (n = 1). Furthermore, the risk-lesions detected were as follows: atypical ductal hyperplasia (n = 3); lobular neoplasia (n = 1), radial scar (n = 1). Of the five patients with personal histories of breast cancer, four were surgically managed, but nonetheless no malignant findings were detected and only one risk-lesion was found. The findings observed in the galactograms according to GICS classification categories are presented in Table 4. The diagnostic performance of technically successful galactography in the detection of neoplastic or risk lesions is presented in Table 5.

**Table 4:** The findings observed in the galactograms according to modified Galactogram Image Classification system (GICS) categories and final histology or follow-up.

Total 146 patients	GICS0	GICS1	GICS2	GICS3	GICS4a	GICS4b	GICS4c	GICS5
**n (%)**	9 (6.2%)	41 (28.1%)	23 (15.8%)	7 (4.8%)	36 (24.7%)	21 (14.4%)	6 (4.1%)	3 (2.1%)
**Surgery**	5 (3.4%)	14 (9.6%)	13 (8.9%)	2 (1.4%)	33 (22.6%)	21 (14.4%)	6 (4.1%)	3 (2.1%)
**follow-up**	4 (2.7%)	27 18.5%	10 (6.8%)	5 (3.4%)	3 (2.1%)	0 (0.0%)	0 (0.0%)	0 (0.0%)
**Malignant**	1	1	0	0	0	1	0	1
**Risk lesion**	0	2	1	0	0	0	1	1
**Papilloma**	2	4	6	1	23	27	4	0

**Table 5:** The diagnostic performance of galactography and MRI in the detection of neoplastic or risk lesions.

Diagnostic performance of galactography				
Sensitivity % (95% CI)	specificity % (95% CI)	OA % (95% CI)	PPV % (95% CI)	NPV % (95% CI)
77.4 (65.60–86.05)	75.7 (64.80–84.03)	76.47 (68.67–82.81)	72.7 (60.96–82.00)	80 (69.18–87.70).
**Diagnostic performance of MRI**				
85.7 (48.68–97.43)	71.4 (45.35–88.28)	76.2 (54.91–89.37)	60 (31.27–83.18)	90.9 (62.27–98.38).

OA, overall accuracy; PPV, positive predictive value; NPV, negative predictive value; CI, confidence interval.

Patients older than 50 yrs. had more often bloody nipple discharge (p = 0.027) and were at increased risk of having an underlying malignant or risk-lesion (4/4 malignant and 4/5 high-risk lesions; 88.9%; p = 0.03). Otherwise, no significant association was found between the presence of papilloma (p = 0.33) or higher galactographic classification (GICS 1,2,3 vs. 4,5 and 6; p = 0.24) in patients >50 yrs. Bloody PND was not significantly associated with malignant or high-risk lesions (p = 0.24) nor with papillomas (p = 0.176).

**Fig 1:**
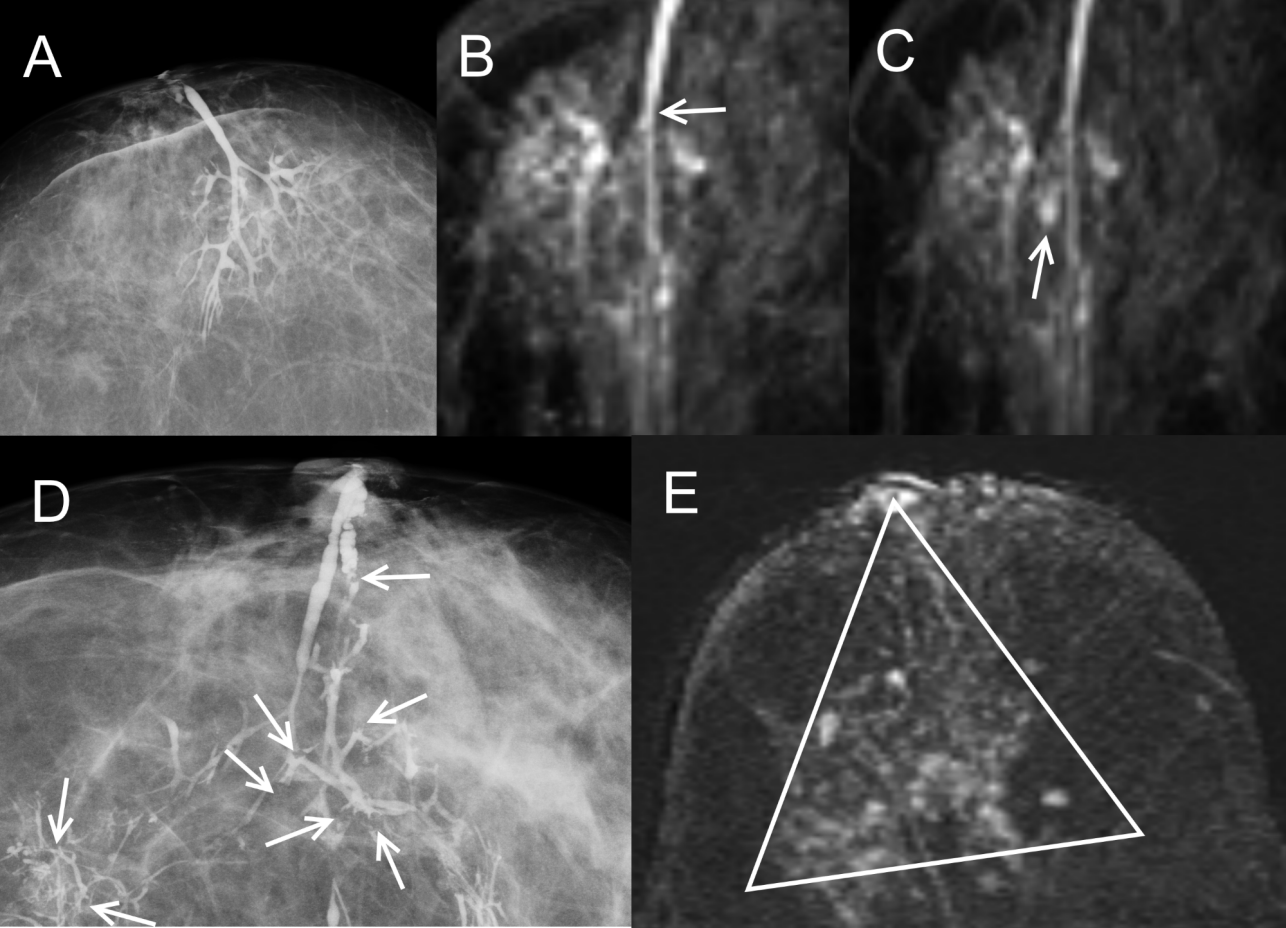
Radiological evaluation of pathological nipple discharge (PND) in two patients. (A) Normal galactogram in 78-years old woman with bloody PND. (B) Same patient’s axial thick slab reformat of the fat-suppressed non-enhanced T1-weighted sequence showing high signal ducts (arrow). (C) same level as 1B after gadolinium enhancement showing a small mass-lesion in the central area (arrow), proved to be a solitary intraductal papilloma at final histology. MRI was useful in detecting and locating the cause of symptoms. (D) Galactogram in 67-years old woman with PND showing multiple microdefects with a moth-eaten appearance, ductal wall irregularities and peripheral stenosis (arrows). (E) Same patient’s axial thick slab reformat of the fat-suppressed gadolinium enhanced T1-weighted sequence showing a segmental non-mass enhancement (triangular shaped with the apex at the nipple) corresponding to the galactogram. The final histology was multiple papillomatosis. MRI before oncoplastic conservative breast surgery did not offer additional information.

MRI examinations were performed in 21 (14.4%) patients. The indications were problem solving in 17 patients (serous PND n = 5, bloody or serosanguineous n = 12) and technical failures of galactography and persisting symptoms in 4 patients (serous PND n = 3) (Fig 1). Of these, one patient (4.8%) was found to have a malignant finding (GICS0, serous PND), two (9.5%) had risk-lesions (GICS 2 and 5, bloody discharge). MRI was interpreted as normal in 10 patients and of these, one DCIS lesion was diagnosed at final histology. In this patient, mammography, US, galactography and MRI were normal at 1-year follow-up, but due to persistent intermittent PND symptoms, this patient underwent surgery. A papilloma and an associated 6 mm low-grade DCIS were diagnosed at final histology. Other findings included one malignant, 2 risk-lesions and 3 papillomas. Furthermore, another 4 patients with MRI findings were surgically treated with benign findings at final histology. Based on these findings, the diagnostic performance of MRI is shown in Table 5.

All patients included had a minimum of 2 years’ follow-up. During follow-up, an invasive carcinoma was detected in the contralateral breast of one patient who previously had PND of benign etiology.

## Discussion

Our diagnostic algorithm reveals that the probability of malignancy in patients with PND is low (2.7%) when CBE, mammography and ultrasound are all negative with respect to the cause of clinical symptoms. This is in line with previous reports that have utilized US in the primary diagnostic protocol. Gray et al reported that the risk of malignancy was 3% when mammography was negative and 0% when additionally subareolar US was also negative [21]. Gelder et al reported a malignancy risk of <2% in a study of 111 patients with US being performed in 103 of these patients [22]. Furthermore, our results indicate that patients younger than 50 yrs. had a negligible probability of having an underlying malignant or a high-risk lesion if CBE mammography and US are all negative.

The vast majority of reports on ND are retrospective. While mammography was usually conducted in the majority of patients with PND, not all studies report whether US or galactography had been performed in all patients and therefore it is difficult to compare outcomes of different studies. In a large study of 306 patients with a negative standard evaluation, Morrogh et al reported that galactography had been performed in 163 patients and it was successful in 139 of them. In the same study, mammography was available and confirmed to be negative in 271/306 (89%) patients and furthermore US was conducted in 44/306 (14%) patients, while both tests were negative in 42 (14%) [13]. Based on our results, it is extremely valuable to conduct a US examination in the first line diagnostic algorithm of PND to minimize the risk of missing a malignancy.

The value of galactography as a second-line diagnostic investigation is debated. It has been criticized as it is an invasive procedure although in fact, it is rather minimally invasive. Nonetheless, galactography does have potential complications. Although perforation of the duct and extravasation of the contrast material can occur, these are self-limiting minor complications with no reported long term sequelae. Furthermore, imaging findings of benign and malignant lesions overlap with no specific signs helping to predict the true histology of these lesions [23,24]. Nevertheless, this is a common situation in diagnostic imaging, not specifically for galactography. The technical failure of galactography is considered to be another limitation of the technique. Our failure rate of 6.2% (9/146) is relatively low compared to the frequently referenced rate of up to 15% [16]. Furthermore, galactography has many advantages: it is a low-cost, readily available technique and in expert hands, easy to perform and interpret. In addition, it might be also speculated that when diagnostic breast imaging is negative, persistently symptomatic patients might be directly offered duct excision without the need of costly additional imaging [25]. Nevertheless, without preoperative mapping with ductography, central duct excision may not result in removal of the actual abnormal ductal tissue or may result in only partial removal of the underlying pathological process resulting in an underestimation of the disease [26].

In an effort to reduce confusion and to standardize the results of galactography, Berná-Serna et al proposed the GICS in a similar fashion to the BI-RADS classification [19]. We further modified this classification to comply with the newest, fifth edition of the BI-RADS. We believe that standardization of galactogram interpretations is needed. However, we were unable to properly evaluate its predictive value due to the very low malignancy rate in the present study. Furthermore, it seems that classification of galactographic findings does not follow the same BIRADS lesion characterization principals of breast findings as most of the high score findings (GICS 4b-5) were benign. Nevertheless, a normal galactogram seems to reliably predict a very low likelihood of malignancy. In this study we had only one associated finding of low grade DCIS in an otherwise normally interpreted galactogram. It might be speculated that this incidental finding is unlikely to develop into a life-threatening disease [27], and it is therefore reasonable simply to follow-up patients with PND whenever CBE, mammography, ultrasound and galactography are all negative. However, breast MRI was reported to be superior to mammography and US in the detection of both malignant and high risk lesions [28], and therefore it can be speculated that it can be used routinely to rule out significant causes of PND after negative conventional imaging. Nevertheless, the sensitivity advantage of MR imaging over conventional imaging was reported to decrease with decreasing nuclear grade of DCIS [29]. Therefore, a negative MRI will not completely rule out low-grade intraductal malignant proliferations, as was also the case in this patient.

Breast MRI has emerged as the most sensitive modality in the detection of breast cancer. MRI was found to be feasible and accurate also in the evaluation of PND. With increasing experience and greater availability of MRI scanners, several reports have described not only the higher sensitivity of MRI as compared to galactography but also its high negative predictive value for malignancy. The EUSOMA guidelines in 2010 evaluated ten papers published on the use of MRI and concluded that there is insufficient evidence of benefit to recommend the routine use of MRI in the clinical context of PND [16]. Since then, a few publications have evaluated the use of MRI in the setting of negative mammography and/or US. A recent meta-analysis evaluated a total of 10 studies and found that MRI exhibited a higher diagnostic performance compared with galactography in the detection of any kind of lesion as well as the high performance of MRI in the detection of cancers in the same patients [30]. The meta-analysis concluded that MRI should be considered in this population if mammography and ultrasound findings are negative. Nevertheless, the MRI study populations were relatively small, and in only few reports had galactography been performed on the whole study population. Thus, in our opinion, the level of evidence remains limited for favouring MRI as a routine secondary investigation. In our study, MRI was performed only as an ancillary problem solving investigation in selected non-consecutive patients. In the diagnosis of the causes of PND, MRI was more sensitive than galactography but both methods showed equal overall accuracy. However, these results should be interpreted cautiously since the values for MRI represent a highly-selective patient population. Nonetheless, whenever galactography is technically unsuccessful, MRI should be considered as an additional ancillary tool to evaluate the possible etiology of the patient’s symptoms.

In patients with PND the clinical symptoms in conjunction with radiological findings guide the type and extent of surgical management. Even if the etiology of PND is confirmed to be definitely benign, the patient might still need to undergo a surgical intervention to relieve the symptoms that affect her quality of life. It is also well known that a negative galactogram does not definitely rule out the presence of some microscopic disease. In our study, 11 patients were found to have papillomas in GICS 1–3 findings and in another 3 patients, a risk-lesion was detected. Nevertheless, this is not a specific issue only for galactography. Sanders et al reported that from 56 patients with a negative MRI (BI-RADS 1–3) seven were consequently diagnosed with high-risk lesions at surgery and in one patient, the MRI had given rise to a false-negative interpretation for malignancy. In the study of Bahl et al, out of 13 patients proceeding to surgery despite negative MRI, one patient had a risk-lesion and another five were found to have intraductal papillomas, one of these with atypia [18]. In this study and in line with previously published results [15,30], the reported specificity of MRI was 71.4%. Yet this result is probably underestimated since MRI was only selectively performed in more complex situations.

The economical aspect of the evaluation of PND is outside the scope of this study. Nevertheless it is worth mentioning that MRI is more expensive than galactography. In our hospital, the list price of galactography is 122€ vs 605€ for MRI. Furthermore, although the reported sensitivity and negative predictive values of MRI in the detection of cancers are high, the specificity is lower (pooled 76%; 95% CI 49–92) [30], and therefore false positive results may result in more additional tests e.g. targeted US and biopsies, including the more expensive MRI-guided interventions all of which increase the total costs.

The retrospective nature of our study is a major limitation. Additionally, although the total number of patients is not particularly small when compared to the published literature, we were not able to fully evaluate the diagnostic accuracy of our modified GICS due to the low prevalence of malignant lesions. MRI was performed in very few patients and therefore, based on our results, no subgroup of patients or galactographic findings that could benefit from MRI can be suggested. Clearly patients with failed galactographic evaluation and persistent symptoms might benefit from MRI. Additionally, it is more appropriate that breast MRI is performed in BRCA mutation carriers and other high risk patients as exposure to ionizing radiation should be minimized in these situations.

## Conclusion

**Fig 2:**
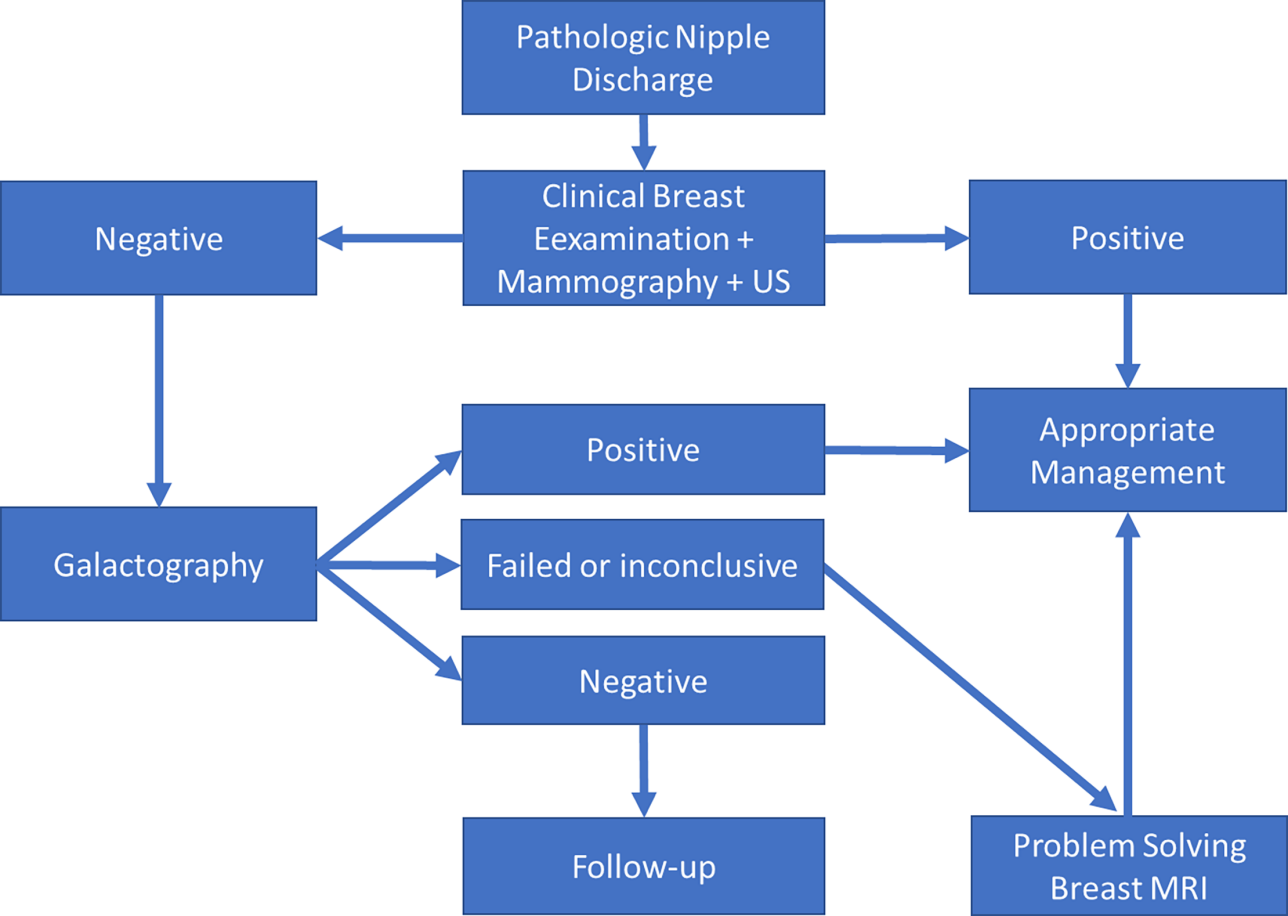
The proposed diagnostic algorithm in the evaluation of pathological nipple discharge.

In PND, a diagnostic algorithm including CBE, mammography and whole breast US is recommended in the first-line investigation, and when negative, we recommend galactography as a second-line procedure Fig 2. The probability of a malignancy is negligible if this diagnostic algorithm is negative. In the current environment of growing medical costs and continuous requirements for cost containment, galactography remains a practical, valuable and cost-effective examination technique. MRI should be included in the diagnostic algorithm of PND only for problem solving in selected populations, e.g. when galactography fails or in the preoperative setting when all other findings remain inconclusive. The inclusion of MRI in the routine evaluation of patients with PND is not justified.

## Clinical practice points

- The diagnostic work-up of pathological nipple discharge remains challenging and investigational strategies are controversial.
- The probability of malignancy in patients with PND is low (2.7%) when CBE, mammography and ultrasound are all negative with respect to the cause of clinical symptoms.
- It is extremely valuable to conduct a US examination in the first line diagnostic algorithm of PND to minimize the risk of missing a malignancy.
- If galactography is technically unsuccessful, MRI should be considered as an additional ancillary tool to evaluate the possible etiology of the patient’s symptoms.
